# Transoral Robotic Surgery in the Management of Submandibular Gland Sialoliths: A Systematic Review

**DOI:** 10.3390/jcm12083007

**Published:** 2023-04-20

**Authors:** Marta Rogalska, Lukasz Antkowiak, Anna Kasperczuk, Wojciech Scierski, Maciej Misiolek

**Affiliations:** 1 Faculty of Medicine, Medical University of Warsaw, 02-091 Warsaw, Poland; 2 Department of Pediatric Neurosurgery, Medical University of Silesia in Katowice, 40-752 Katowice, Poland; lukaszantkowiak7@gmail.com; 3 Faculty of Mechanical Engineering, Institute of Biomedical Engineering, Bialystok University of Technology, 15-351 Bialystok, Poland; a.kasperczuk@pb.edu.pl; 4 Department of Otorhinolaryngology and Oncological Laryngology, Faculty of Medical Sciences in Zabrze, Medical University of Silesia in Katowice, 41-800 Zabrze, Poland; wojciech.scierski@sum.edu.pl (W.S.); maciej.misiolek@sum.edu.pl (M.M.)

**Keywords:** sialolithotomy, sialendoscopy, robot-assisted, sialolithiasis, submandibular stones, lingual nerve

## Abstract

This study aimed to systematically review the literature to determine the efficacy and safety of transoral robotic surgery (TORS) in the management of submandibular gland (SMG) sialolithiasis. PubMed, Embase, and Cochrane were searched for English-language articles evaluating TORS in the management of SMG stones published up to 12 September 2022. Nine studies with a total of 99 patients were included. Eight patients underwent TORS followed by sialendoscopy (TS); 11 patients underwent sialendoscopy followed by TORS and sialendoscopy (STS); 4 patients underwent sialendoscopy followed by TORS only (ST); and 4 patients underwent TORS without sialendoscopy (T). The mean operative time amounted to 90.97 min. The mean procedure success rate reached 94.97%, with the highest for ST (100%) and T (100%), followed by the TS (95.04%) and STS (90.91%) variants. The mean follow-up time was 6.81 months. Transient lingual nerve injury occurred in 28 patients (28.3%) and was resolved in all of them within the mean of 1.25 months. No permanent lingual nerve injury was reported. TORS is a safe and effective management modality for hilar and intraparenchymal SMG sialoliths, with high procedural success in terms of successful sialolith removal, SMG preservation, and reduced risk of permanent postoperative lingual nerve damage.

## 1. Introduction

Sialolithiasis represents the most common cause of obstructive salivary gland disorders [1]. While postmortem studies indicate a 0.115% prevalence of sialoliths in the general population, their clinical (symptomatic) prevalence amounts to 0.45% [1,2,3]. Most salivary stones (as high as 80–90% of cases) affect the submandibular gland (SMG), with a preferential location in the distal third of the Wharton’s duct, at the hilum or in the hilo-parenchymal area of the SMG [1].

The removal of large proximal or hilo-parenchymal SMG sialoliths has traditionally been managed by means of transcervical sialoadenectomy, which carries a significant risk to the marginal mandibular nerve and might lead to an aesthetically unappealing scar [1,4]. With the advancement of sialendoscopy, a combined approach (CA) technique incorporating sialendoscopy and transoral sialolithotomy has enabled SMG preservation with a procedure success rate ranging from 90% to 100% [5,6,7,8,9,10,11]. Notably, transoral duct surgery with interventional sialendoscopy, as well as intraductal shock wave lithotripsy (ISWL) can be performed in local anesthesia, the latter of which has reported success rates above 90% [12,13,14].

Despite being superior to the previous non-gland-sparing modalities, the CA sialolithotomy poses several challenges, which are magnified the closer the SMG stone is to the hilum. The higher risk of lingual nerve damage due to its intimate relationship with Wharton’s duct near its exit point at the SMG hilum contributes to the 2% rate of permanent tongue paresthesia reported after the CA procedure [15]. Furthermore, poor visualization and limited space for instrumentation, amplified in the presence of unfavorable anatomy and physical features such as obesity, reduced mouth opening, and prominent teeth, represent additional considerable drawbacks of the CA technique [16].

Recently, the application of robotic technology in the treatment of various head and neck disorders (obstructive sleep apnea, and pathologies involving the thyroid, parathyroid, oropharynx, hypopharynx, and supraglottis [17]) has favored the spread of this procedure for the removal of proximal hilar submandibular duct sialoliths. Since the initial experiences with robot-assisted SMG sialolithotomy, as well as its outcomes and advantages compared to the CA technique, have been reported, the purpose of the present study was to systematically review the literature to determine the efficacy and safety of transoral robotic surgery (TORS) in the management of SMG sialolithiasis.

## 2. Materials and Methods

### 2.1. Study Guidance

The review was conducted according to the PRISMA (Preferred Reporting Items for Systematic Reviews and Meta-Analyses) guidelines [18]. The study protocol was registered with the International Platform of Registered Systematic Review and Meta-analysis Protocols (INPLASY) under the number INPLASY202330068 [19].

### 2.2. Search Strategy and Criteria

The PubMed, Embase, and Cochrane databases were searched by two authors (M.R. and L.A.) independently for English-language full-text papers published from inception until 16 September 2022. Comprehensive electronic search strategies included terms for submandibular gland sialoliths (“submandibular” OR “salivary” OR “gland” OR “sialolithiasis” OR “sialolith” OR “megalith” OR “stone”) AND terms for operative technique (“sialolithotomy” OR “sialoendoscopy” OR “sialendoscopy” OR “transoral”) AND terms for robotic assistance (“robot” OR “robotic” OR “robot-assisted” OR “robotic assisted”).

After duplicate removal, all studies were screened by two authors (M.R. and L.A.) independently, based on the title and the abstract. Inclusion criteria comprised clinical studies, case series, and case reports evaluating TORS in the management of submandibular gland stones. Contrarily, publications with an unrelated topic as well as conference papers, review articles, commentaries, and letters to the editor, were excluded. Additionally, the reference lists in all preselected articles were screened for further relevant papers.

### 2.3. Eligibility Criteria

The study was found eligible if it described the application of robot-assisted sialolithotomy (RAS) in the removal of the submandibular gland sialoliths.

### 2.4. Data Extraction and Analysis

From the included studies, the following data were extracted: first author and publication year, study design, number of patients, sialolith location(s), sialolith size(s), used robotic surgical system, variation of TORS-assisted sialolithotomy (i.e., (1) TORS immediately followed by sialendoscopy (TS); (2) sialendoscopy immediately followed by TORS and subsequent sialendoscopy (STS); (3) sialendoscopy immediately followed by TORS only (ST); TORS without sialendoscopy (T)), procedure success rate, procedure duration, intraoperative complications, postoperative complications, and time until symptom resolution. If RAS consisted of more than one step (i.e., TS, STS, ST), all of them were performed within the same surgical procedure. Procedure success was defined as a successful sialolith removal with submandibular gland preservation and absence of symptom recurrence at the latest available follow-up. In order to calculate the weighted averages of all available quantitative parameters, weights were selected proportionally to the sample size.

## 3. Results

### 3.1. Study Selection

**Figure 1:**
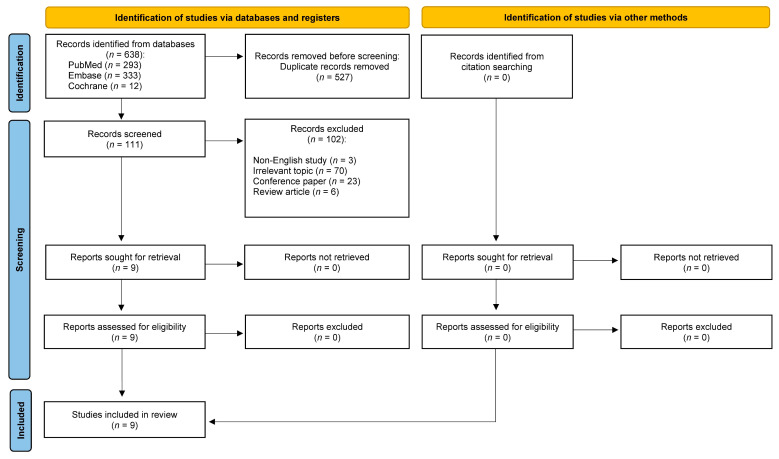
PRISMA flowchart of the medical database search strategy.

The literature search yielded 638 articles, including 293 from PubMed, 333 from Embase, and 12 from Cochrane. After the removal of 527 duplicate records, 111 studies were screened. Three non-English studies and 70 articles with an irrelevant topic were excluded, as well as 23 conference papers and 6 review articles. The remaining nine articles [4,15,16,20,21,22,23,24,25] were found eligible and included in the further analysis. Figure 1 shows the entire literature selection process.

### 3.2. Study Characteristics

The included studies involved a total of 99 patients. Eight patients from four studies [4,15,20,22] underwent TORS followed by sialendoscopy (TS). In eleven patients from two studies [4,23], sialendoscopy followed by TORS and sialendoscopy (STS) was performed. Four patients from two studies [4,16] underwent sialendoscopy followed by TORS only (ST), whereas in the remaining four patients from three studies [21,24,25], TORS without sialendoscopy (T) was performed. Complete study characteristics are presented in Table 1.

### 3.3. Sialolith Size

Sialolith size was evaluated in all nine studies [4,15,16,20,21,22,23,24,25]. The mean sialolith size amounted to 11.46 mm (range 4–28 mm).

### 3.4. Aim of Sialendoscopy

Sialendoscopy prior to the sialolith removal was performed to facilitate sialolith localization in 15 patients [4,16,23]. In Wen et al.’s study [4], a sialendoscopy-first approach (ST or STS) was selected in case of non-palpable or multiple sialoliths. In a patient described by Vergez et al., sialendoscopy allowed the identification of a hilar sialolith impacted beyond proximal ductal stenosis [23]. In 91 patients [4,15,20,22,23], after the successful sialolith removal, the ductal system was explored with a sialendoscope to ensure the submandibular duct patency by identifying any additional sialoliths, remaining stone fragments, or areas of ductal stenosis.

### 3.5. Procedure Duration Time

Procedure duration time was reported in all nine studies [4,15,16,20,21,22,23,24,25]. The mean operative time amounted to 90.97 min (range 13–143 min) and was the shortest for the T (43.33 min), followed by the TS (76.03 min), STS (177.27 min), and ST (189.75 min) techniques.

### 3.6. Procedure Success Rate

Procedural success was described in all nine studies [4,15,16,20,21,22,23,24,25]. The mean procedure success rate reached 94.97%, with the highest for the ST (100%) and T (100%), followed by the TS (95.04%) and STS (90.91%) variants.

### 3.7. Follow-Up Time

The duration of the follow-up was reported in seven studies [4,15,20,21,22,24,25]. The mean follow-up time was 6.81 months (range 0.35–65.53 months).

### 3.8. Complications

Transient lingual nerve injury occurred in 28 patients (28.3%) from five studies [4,15,20,22,24] and resolved in all of them within the mean of 1.25 months (range 0.5–2.8 months). No permanent lingual nerve injury was described in the included studies.

## 4. Discussion

The initial implementation of robotic assistance in head and neck surgery has concerned predominantly oncologic indications since it reduced hospitalization length and enabled access to tumors in challenging anatomic locations [26]. However, numerous authors have recently emphasized the benefits of incorporating TORS in the management of non-oncologic pathologies located in areas with poor operative exposure, including large proximal, hilar, or hilo-parenchymal SMG sialoliths [4,15,20].

The advantages of robot-assisted surgery might result from the magnified three-dimensional view of the surgical field, which allows the surgeon to have an accurate anatomical delineation and enhanced perception of the depth of the oral floor, lingual nerve, Wharton’s duct, and hilo-parenchymal SMG region [15,20,21]. Furthermore, the heightened operative visualization facilitates the use of smaller incisions, allows the better identification of vital structures (such as the lingual nerve), and enables decreased manipulation of Wharton’s duct. The functional preservation of the main submandibular duct simplifies sialendoscopic access through its natural ostium in case of residual microliths [21]. Additionally, due to the medicolegal ramifications of the lingual nerve injury, the video documentation of an intact nerve, available by means of the RAS procedure, is crucial, even if, at certain stages of the procedure, its mobilization is unavoidable [4]. Moreover, since all surgical steps are visible to the whole operating room staff, the crowding around the operating space is reduced, and the use of the robotic unit can serve as an excellent teaching tool for residents and medical students [4,16].

Increased dexterity and precision due to the 360° range of motion provided by the robotic instrumentation result in improved tissue manipulation, less unnecessary trauma to the local structures, and a safer dissection of the lingual nerve and Wharton’s duct at the SMG hilum. Razavi et al. suggested that the abovementioned advantages might partially prevent postoperative ductal scarring and stenosis, which may ultimately enable the avoidance of symptom recurrence and the necessity of reoperation [15]. Furthermore, contrarily to the CA technique, RAS allows for the greater involvement of a surgical assistant without compromising the operative field visually or spatially [15]. Thus, the assistant surgeon can simultaneously perform the suction, tissue traction, and push-up of the SMG from the neck in order to better expose the parenchyma in the oral floor [21].

Another significant but frequently overlooked advantage of robotic surgery is the benefit of improved surgical ergonomics [27]. A comfortable seated position and decreased prolonged neck strain might reduce the frequency of work-related musculoskeletal disorders among ENT specialists and ultimately lead to the increased career length of a head and neck surgeon.

The numerous abovementioned technical advantages of RAS compared to the CA technique might contribute to the higher success rate of the robot-assisted approach (94.97% vs. 75–87% [15,28], respectively). Notably, of the five patients in our review where RAS was unsuccessful, three individuals experienced symptom recurrence [4]. Two of them required sialendoscopy for recurrent sialoliths removal; in one of them, SMG excision was ultimately necessitated, during which frank purulence, SMG fibrosis, and a 5 mm intraparenchymal stone were discovered [4]. Of the remaining two patients, in one individual, the sialolith could not be localized on sialendoscopy due to the extensive scarring of the surgical field [20]. This prompted SMG excision, which revealed three sialoliths within the SMG parenchyma and the proximal Wharton’s duct [20]. In the other patient, who suffered from frequent sialadenitis secondary to sialolithiasis, significant inflammation and fibrosis of the SMG and surrounding tissues made the localization of the sialolith unfeasible, and SMG removal was eventually required [20]. Given these failures, the robot-assisted technique might be less successful in the case of deep parenchymal localization of the sialoliths and considerable SMG fibrosis resulting from chronic inflammation.

Additionally, due to the significant discrepancies in sample sizes between the applied TORS variations (T, TS, ST, STS), care must be taken when interpreting the differences in their success rates. Although the success rates of the ST and T techniques (amounting to 100%) were higher than the success rates of the TS variations (95.04%), the ST and T groups were considerably smaller than the TS sample (4 patients vs. 80 patients, respectively). With larger sample sizes in the ST and T groups, their actual success rate could noticeably decrease, thus reducing the difference between the effectiveness of each technique. Furthermore, the success rates of various TORS modifications should not be juxtaposed since each management method was applied for specific indications (single vs. multiple, palpable vs. non-palpable, hilar vs. hilo-parenchymal sialoliths). Generally, palpable sialoliths and those ≥5 mm on imaging were treated by the TORS-first approach, whereas, in the case of multiple or unpalpable SMG stones, the sialendoscopy-first approach was selected, similarly to the algorithm proposed by Quiz et al. [4,5]. Based on the results of our review, we state that all techniques proved to be highly effective, taking into account the indications for their implementation. Nonetheless, randomized control trials with patients anonymously assigned to each group (ST, STS, TS, or T) regardless of the sialolithiasis characteristics are necessary to compare the success rates of TORS variations.

Despite the often-cited belief that the employment of the robot in SMG sialolithotomy increases the operative time, our analysis revealed the mean procedure time amounted to 90.97 min, which is similar to or slightly shorter than the average of 90 to 113 min for the conventional CA technique [27,29]. Nonetheless, due to the scarcity of the literature describing CA procedure times and the fact that the available reports date back to the time when CAS was a more novel procedure, the actual CA operative time could have decreased with greater surgical experience. 

Additionally, our results suggest a lower incidence of permanent lingual nerve damage with RAS compared to the CA technique (0% vs. 2%, respectively). Importantly, the literature regarding the presence of lingual nerve injury after sialolith removal via the CA technique includes both patients with hilar and ductal SMG sialoliths, the latter of which are not as intimately related to the lingual nerve as those in the hilar location. Nevertheless, even considering the higher inherent risk to the lingual nerve in our review due to the hilar or intraparenchymal localization of all sialoliths, permanent lingual nerve damage was omitted in all cases. 

Despite many advantages of robotic assistance in SMG sialolithotomy, the lack of tactile feedback and the necessity of greater reliance on visual cues constitute one of its significant limitations [15,20,23]. However, this disadvantage might be partially mitigated through intraoperative stone palpation by the assistant surgeon and due to the fixed position of most hilar SMG sialoliths [15]. Tissue mobility might be interpreted as a haptic sense, but only by an experienced robotic surgeon; therefore, the incorporation of the preoperative ultrasonography and Cone Beam CT might be mandatory to successfully pursue the excision of purely unpalpable parenchymal SMG sialoliths [5,30,31].

Furthermore, according to our analysis, the mean sialolith size was greater than that reported in the literature regarding the sialolith excision via the non-robotic transoral technique [5,7,8,10,11,32,33,34]. With the increase in the sialolith size, the necessity of tactile feedback diminishes, which facilitates the robotic removal of SMG stones. Contrarily, smaller sialoliths impose the importance of stone palpation, which cannot be provided by RAS.

Notably, the possible traumatic mechanical effect of robotic instruments during RAS might contribute to the high rate of postsurgical transient lingual nerve injury (28.3%) in our review. Although our results are higher than those reported in the literature regarding the CA technique [7,9,33,35,36], patients from the included studies were considered to suffer from transient lingual nerve injury, even if the lingual paresthesia remained very subtle. Additionally, all patients in our review were treated with the Da Vinci Si and SP robot (Intuitive Surgical Inc., Sunnyvale, CA, USA), or Flex Robotic system (Medrobotics Inc., Raynham, MA, USA). Notably, Da Vinci Si (Intuitive Surgical Inc., Sunnyvale, CA, USA) has recently been replaced by the more advanced Da Vinci Xi robotic system (Intuitive Surgical Inc., Sunnyvale, CA, USA). The difference in the instrument sizes between the robotic systems might influence their handling and associated tissue damage during the procedure [37]. We hypothesize that the transient lingual nerve injury rate could be decreased by the wider application of RAS, which would improve the learning curve of head and neck surgeons.

Furthermore, RAS remains a reasonable approach mainly for large, deeply located sialoliths, and when unfavorable conditions such as pharyngeal reflex are present. In challenging anatomic conditions (e.g., markedly reduced mouth opening), RAS, as with other transoral approaches, might not be technically feasible. Another significant disadvantage of RAS is the necessity of performing the surgery under general anesthesia.

Finally, a considerable drawback of robotic assistance is the limited availability of the device in rural areas. Additionally, significant costs associated with the RAS procedure limit its wide applicability across multiple institutions. Conversely to tertiary medical centers, where this technology is utilized in multiple surgical specialties, smaller hospitals with a lower case volume might find this technology financially disadvantageous [20,23].

### Limitations

Our systematic review comprises mainly case series and non-randomized, retrospective, single-center studies with limited sample sizes; thus, we advocate caution in interpreting the results. Moreover, the exclusion of non-English-language papers could have restricted the already scarce literature describing RAS in the management of patients with SMG sialolithotomy. Additionally, although the mean follow-up time in our review amounting to 6.81 months is long enough to capture postoperative complications such as lingual nerve damage, it might be insufficient to describe the actual rate of SMG sialolithiasis recurrence.

## 5. Conclusions

RAS is a safe and effective management modality for hilar and intraparenchymal SMG sialoliths, with a high procedural success in terms of successful sialolith removal and SMG preservation, and a vastly reduced risk of permanent postoperative lingual nerve damage. Future prospective studies with expanded RAS cohorts and longer follow-up times are highly warranted to precisely define the extent of RAS utility and reliability in the management of patients with SMG sialoliths.

## Figures and Tables

**Table 1:** Characteristics of studies included in the systematic review.

First Author (year)	No. of Patients	Sialolith Size, Mean (Range) (mm)	Sialolith Location	Robotic Surgical System	Procedure Success Rate (%)	Procedure Duration, Mean (Range) (min)	Follow-Up Time, Mean (Range)	Transient Lingual Nerve Injury (%)	Duration of Transient Lingual Nerve Injury, Mean (Range)	Permanent Lingual Nerve Injury (%)
**ST**										
Wen (2021) [4]	3	8.7	hilar or intraglandular	da Vinci Si or da Vinci SP (Intuitive Surgical Inc., Sunnyvale, CA, USA)	100%	213 (157–283)	2.8 months	-	-	0%
Walvekar (2011) [16]	1	19	hilo-parenchymal	da Vinci Si (Intuitive Surgical Inc., Sunnyvale, CA, USA)	100%	120	-	0%	-	0%
**STS**										
Wen (2021) [4]	10	8.6	hilar or intraglandular	da Vinci Si or da Vinci SP (Intuitive Surgical Inc., Sunnyvale, CA, USA)	90.0%	190 (88–301)	12.9 months	-	-	0%
Vergez (2021) [23]	1	10	hilar	da Vinci Si (Intuitive Surgical Inc., Sunnyvale, CA, USA)	100%	50	-	0%	-	0%
**TS**										
Razavi (2016) [15]	22	12.3 (5–20)	hilar	da Vinci Si (Intuitive Surgical Inc., Sunnyvale, CA, USA)	100%	67 (38–143)	14 (5–25) months	18%	2.5 (2–3) weeks	0%
Tampio (2021) [20]	33	8.9 (5–20)	hilar	da Vinci Si (Intuitive Surgical Inc., Sunnyvale, CA, USA)	94%	62 (13–111) *	19 (14–21) days	15.1%	54 (30–84) days	0%
Wen (2021) [4]	24	14.5	hilar or intraglandular	da Vinci Si or da Vinci SP (Intuitive Surgical Inc., Sunnyvale, CA, USA)	91.7%	103 (57–184)	7.2 months	-	-	0%
Frost (2020) [22]	1	19	hilar	da Vinci SP (Intuitive Surgical Inc., Sunnyvale, CA, USA)	100%	-	3 months	100%	-	-
**T**										
Capaccio (2019) [21]	1	15	hilo-parenchymal	da Vinci Si (Intuitive Surgical Inc., Sunnyvale, CA, USA)	100%	55	3 months	0%	-	0%
Capaccio (2019) [21]	1	8	hilo-parenchymal	da Vinci Si (Intuitive Surgical Inc., Sunnyvale, CA, USA)	100%	45	3 months	0%	-	0%
Capaccio (2021) [24]	1	15	hilo-parenchymal	Flex Robotic System (Medrobotics Inc., Raynham, MA, USA)	100%	130 (bilateral)	3 months	100%	1 month	0%
Capaccio (2022) [25]	1	25	hilo-parenchymal	Flex Robotic System (Medrobotics Inc., Raynham, MA, USA)	100%	30	3 months	0%	-	0%

Legend: TORS, transoral robotic surgery; TS, TORS followed by sialendoscopy; STS, sialoendoscopy followed by TORS and sialendoscopy; ST, sialoendoscopy followed by TORS; T, TORS without sialendoscopy. * calculated based on 32 patients since one patient underwent TS on the left and an additional sialendoscopy with wire basket retrieval of a contralateral submandibular gland stone. This patient’s procedure time was excluded from the analysis.

## Data Availability

The data generated during this study are available within the article. Datasets analyzed during the current study preparation are available from the corresponding author on reasonable request.
